# Severe Pneumonia Caused by Respiratory Syncytial Virus and Adenovirus in Children from 2 to 24 Months at Children’s Hospital 1 in Ho Chi Minh City, Vietnam

**DOI:** 10.3390/v16030410

**Published:** 2024-03-07

**Authors:** Suong Thi Thu Nguyen, Tuan Anh Tran, Giau Van Vo

**Affiliations:** 1 School of Medicine, Vietnam National University—Ho Chi Minh City (VNU-HCM), Ho Chi Minh City 710000, Vietnam; 2 Vietnam National University—Ho Chi Minh City (VNU-HCM), Ho Chi Minh City 700000, Vietnam; 3 Children’s Hospital 1, Ho Chi Minh City 710000, Vietnam; 4 Research Center for Genetics and Reproductive Health (CGRH), School of Medicine, Vietnam National University, Ho Chi Minh City (VNU-HCM), Ho Chi Minh City 70000, Vietnam

**Keywords:** respiratory syncytial virus (RSV), adenovirus (Adv), severe pneumonia, multiplex real-time PCR, Nasal Continuous Positive Airway Pressure (NCPAP)

## Abstract

In Vietnam, due to the lack of facilities to detect respiratory viruses from patients’ specimens, there are only a few studies on the detection of viral pathogens causing pneumonia in children, especially respiratory syncytial virus (RSV) and adenovirus (Adv). Here, we performed a cross-sectional descriptive prospective study on 138 children patients from 2 to 24 months old diagnosed with severe pneumonia hospitalized at the Respiratory Department of Children’s Hospital 1 from November 2021 to August 2022. The number of patients selected in this study was based on the formula n = ([Z(1 − α/2)]2 × P [1 − P])/d2, with α = 0.05, *p* = 0.5, and d = 9%, and the sampling technique was convenient sampling until the sample size was met. A rapid test was used to detect RSV and Adv from the nasopharyngeal swabs and was conducted immediately after the patient’s hospitalization. Laboratory tests were performed, medical history interviews were conducted, and nasotracheal aspirates were collected for multiplex real-time PCR (MPL-rPCR) to detect viral and bacterial pathogens. The results of the rapid test and the MPL-rPCR in the detection of both pathogens were the same at 31.9% (44/138) for RSV and 8.7% (7/138) for Adv, respectively. Using MPL-rPCR, the detection rate was 21% (29/138) for bacterial pathogens, 68.8% (95/138) for bacterial–viral co-infections, and 6.5% (9/138) for viral pathogens. The results showed few distinctive traits between RSV-associated and Adv-associated groups, and the Adv group children were more prone to bacterial infection than those in the RSV group. In addition, the Adv group experienced a longer duration of treatment and a higher frequency of re-hospitalizations compared to the RSV group. A total of 100% of Adv infections were co-infected with bacteria, while 81.82% of RSV co-infected with bacterial pathogens (*p* = 0.000009). This study might be one of the few conducted in Vietnam aimed at identifying viral pathogens causing severe pneumonia in children.

## 1. Introduction

Community-acquired pneumonia is a common respiratory disease affecting all ages, especially children under 5 years old. The incidence and hospitalization rate due to pneumonia remains at a high level, given that it is the leading cause of death in children, especially in low-income countries like Cambodia, Laos, Vietnam, China, and the Philippines [1]. The etiology of pneumonia encompasses various infectious organisms. However, viruses account for a significant proportion, as shown by the study by Kouni et al. [2], in which the co-infection of viruses in children with acute respiratory infection accounted for 42.5%. According to numerous studies and the literature, viruses were the most common cause of acute lower respiratory tract infections. Notably, respiratory syncytial virus (RSV) was particularly prevalent in infants, especially those under 6 months of age. In addition, other viruses such as adenovirus (Adv), influenza, parainfluenza, rhinovirus, and human metapneumovirus were also important causes, with Adv being the second most common after RSV [2,3]. Rodríguez-Martínez et al. from 2009 to 2011 documented 2.267 children with lower respiratory infections [3], of which 87,8% were identified as being caused by RSV, while 9% were attributed to Adv infection; 3.1% were found to be the result of both RSV and Adv infection. In some cases, Adv infections resulted in a more severe illness than RSV, leading to life-threatening and prolonged pneumonia and necessitating admission to the intensive care unit for respiratory support [4]. In addition, Adv is highly contagious and there is no preventive vaccine available to mitigate its spread at present. Hence, we carried out this study with the primary aim of assessing the occurrence of viral and bacterial–viral co-infection in children aged 2 months to 24 months of age with severe community-acquired pneumonia requiring oxygen therapy in the emergency room of the respiratory department of Children’s Hospital 1. Additionally, this study sought to examine the characteristics of clinical, subclinical, and microbiological characteristics along with treatment outcomes of severe viral, bacterial, and bacterial–viral co-infection pneumonia in this population.

## 2. Methods

### 2.1. Study Design and Population

This is a cross-sectional descriptive prospective study on 138 children patients from 2 to 24 months old diagnosed with severe pneumonia hospitalized at the Respiratory Department of Children’s Hospital 1 from November 2021 to August 2022. The inclusion criteria were (i) age from 2 to 24 months old; (ii) clinical signs including cough, shortness of breath, tachypnea according to age, chest indrawing, and indications for oxygen (WHO 2016); and (iii) parenchymal injury detected by a chest radiograph. On the other hand, following the guidelines for the diagnosis and management of bronchiolitis by the WHO in 2016, we excluded children who had respiratory failure due to being diagnosed with bronchiolitis. Some specific characteristic features include a viral upper respiratory prodrome followed by increased respiratory effort (e.g., tachypnea, nasal, flaring, chest retractions) and wheezing and/or crackles in children younger than two years of age, as well as chest radiographs that are not damaging to lung parenchyma. The pediatric patients infected with COVID-19 confirmed by a rapid test and/or PCR, or patients whose nasal tracheal aspirate (NTA) could not be collected or whose NTA could not meet the Bartlett score [5] to indicate the high quality of the expectorated sputum based on the Gram stain smear of the specimen at the laboratory right after receiving the sample, or patients who had received intravenous antibiotics within 24 h before admission were excluded from this study. The total number of the patients selected in this study was based on the formula n = ([Z_(1−α/2)_]^2^ × P [1 − P])/d^2^, with α = 0.05, *p* = 0.5, and d = 9%; then, *n* = 118. The sampling technique used in this study was convenient sampling until the sample size was met.

All pediatric patients who met the mentioned criteria were interviewed for medical history and clinical examination. Laboratory tests, including complete blood count, CRP, chest X-ray, and nasopharyngeal swab to quickly detect respiratory syncytial virus (RSV) and adenovirus (Adv), were carried out. The NTA samples were collected within 24 h of admission. The chest radiograph was reviewed by the head physician of the radiology department. Disease progression, laboratory results, and antibiotic therapy were recorded. To analyze NTA samples, 1 mL of each sample collected from patients was sent to the laboratory for multiplex real-time PCR (MPL rPCR) testing to detect the microorganism pathogens with the protocol and materials, as in a previous study [6,7]. In addition, the collected NTA samples were also tested for RSV and Adv antigens using the “FUJI DRI-CHEM IMMUNO AG RSV/Adv” system based on the immuneoelectrophoresis principle (95% sensitivity and 100% specificity compared to standard PCR). Patients with RSV and Adv pneumonia were characterized in Table 1.

### 2.2. Statistical Analysis

The collected data were processed using SPSS 20.0 software for subsequent statistical analysis. Categorical variables were compared using χ^2^ or Fisher’s exact test. The statistical significance was defined as *p* < 0.05. Data are expressed as the number of cases and the percentage.

### 2.3. Ethics Statement

This study was approved by the Ethics Committee of Children’s Hospital 1 in Ho Chi Minh City on 7 December 2022 (No. 269/GCN-BVND1). Stringent measures were in place to ensure patient privacy and data confidentiality. Personal identifying information was anonymized during data analysis, and all research procedures adhered to the ethical guidelines and regulations applicable in Vietnam. The medical record information of the patients participating in this study was used for research purposes only and not for any other purpose.

## 3. Results

From November 2021 to August 2022, there were 138 pediatric patients from 2 to 24 months old admitted to the Respiratory Department who met the inclusion criteria. Among these patients, 93 were boys and 33 were girls; the mean age was 7.39 months old with the youngest being 2 months old and the oldest being 23 months old. The most common age range was from 2 to 6 months old (59.4%), followed by 6 to 12 months old (21%) and over 12 months old (18.8%). There were 32 cases (23.2%) of low birth weight or preterm births and 29 cases (21%) that had postpartum respiratory failure.

All of these patients underwent rapid tests for RSV and Adv. The results from the rapid test showed seven cases positive for Adv, forty-four cases positive with RSV, and eighty-seven cases negative with both Adv and RSV. The NTA for multiplex real-time PCR testing was taken within the first 24 h of admission and sent quickly to the laboratory. The results of the MPL rPCR in the detection of Adv and RSV were completely consistent with those of the rapid test. The MPL rPCR results also showed that there were twenty-nine cases positive for bacterial pathogens (21%), ninety-five cases positive for bacterial and viral pathogens (68.8%), nine cases positive for viral pathogens (6.5%), and five cases negative for both bacterial and viral pathogens (3.7%). Table 2 shows the results of the MPL rPCR in the detection of bacterial and viral pathogens in 138 NTA samples.

Table 2 data indicate that among the bacterial pathogens, *S. pneumoniae* was the pathogen with the highest detection ratio (49.8%). Among the atypical bacterial pathogens, *Chlamydia trachomatis* was detected with a ratio of 13.1%, higher than that of *Mycoplasma pneumoniae* (5.7%). RSV exhibited the highest detection ratio (31.9%) among the viral pathogens. CMV had a high detection ratio of 25.4%, although this viral pathogen is often regarded as the etiological pathogen of pneumonia in the immunocompromised host; CMV is also considered a potential pathogen of severe pneumonia in children in non-HIV-infected children [8].

In order to analyze the differences between severe pneumonia associated with RSV and Adv, the related laboratory and treatment characteristics were also reported and shown in Table 3 and Table 4.

In Table 3, the obtained data show that (i) a white blood cell (WBC) count of more than 15,000 cell/mm^3^ was mainly seen in severe pneumonia associated with Adv; however, in the RSV group, WBC count was ≤15,000 cell/mm^3^, and the difference was statistically significant with *p* = 0.047 and χ^2^ = 6.003. (ii) Severe pneumonia cases with C-reactive protein (CRP) more than 35 mg/L were encountered mainly in the Adv group, while CRP ≤ 35 mg/L was mainly in the RSV group, and the difference was statistically significant with *p* = 0.012 and χ^2^ = 8.795. (iii) Elevated liver enzymes were mainly observed in the Adv group (57.1%).

The obtained data in Table 4 show that (i) most patients in the RSV group were treated in less than 2 weeks, while most of the Adv patients were hospitalized for over 30 days, and the difference was statistically significant with *p* = 0.002 and χ^2^ = 18,375. (ii) Some patients in both groups were re-hospitalized once; however, readmission more than three times was only recorded in the Adv group, including one case that progressed to PIBO (postinfectious bronchiolitis obliterans), and the difference was statistically significant with *p* = 0.0021 and χ^2^ = 37,059.

Why did the two groups of children with severe pneumonia exhibit differences in laboratory findings and treatment outcomes in which the Adv group was more prone to bacterial infection (high white blood cell count, high CRP, increased liver enzymes), required a longer duration of treatment, and experienced more frequent re-hospitalizations than the RSV group? To answer this question, it was necessary to analyze the bacterial co-infection of the two groups. The analyzed results showed that in the RSV group, there were 36 cases of bacterial pathogen co-infection, accounting for 81.82% (36/44), while in the Adv group, 100% (7/7) were co-infected with the bacterial pathogens. Although the number of cases in the Adv group in this study was relatively low (only seven cases), with the statistical analysis using the binomial test, this difference was statistically significant with *p* = 0.000009.

## 4. Discussions

The present study shows that RSV and adenoviruses are significant causes of acute severe pneumonia in infants and young children in low- and middle-income countries, including Vietnam. Additionally, the findings of the present study suggest that the Adv group children were more prone to bacterial infection than those in the RSV group. Also, patients co-infected with other pathogenic bacteria were more frequently observed in both groups. Of all the viral infections, RSV was the most often detected, affecting 31.9% of patients; rhinovirus was detected in only 8.7% of cases. According to a prior Vietnamese study, 632 infants under the age of two who had community-acquired pneumonia had up to 48% of RSV and 6% of Adv [9]. Compared to our study, which did not include the rainy season, this one may have included two RSV infection episodes (the rainy season) from May 2009 to December 2010. The RSV detection ratio in our study was greater than that of a study conducted on 1082 hospitalized children with lower respiratory tract infections between April 2010 and May 2011, which revealed a 23.8% RSV detection rate [10]. Benjamin M. Althouse et al. observed 15.2% (455/2998) plus influenza A virus 6.1% and rhinovirus 19% in a distinct study conducted between 2007 and 2012 on children hospitalized for acute respiratory virus infection in the city of Nha Trang, located in central Vietnam. Nevertheless, the detection ratio of Adv in this investigation was a mere 2.9% [11]. Naturally, because of the high humidity during the rainy season, the highest rate of infectious respiratory infections was seen. Given that Nha Trang, which is directly overlooking the sea, has a more temperate dry climate with a shorter rainy season than Ho Chi Minh City, which is located further inland and experiences intense heat and humidity during the rainy season, the differences are most likely due to the different geographic areas with different climates.

In our study, the rates of severe RSV-associated and advanced pneumonia groups requiring mechanical ventilation (NTT, CPAP, oxygen/cannula, etc.) were comparable. On the other hand, the Adv group saw a greater rate of re-hospitalization than the RSV group. These results are consistent with a number of evaluations of the literature that indicated that RSV was the most common virus responsible for lower respiratory tract infections in babies, with Adv coming in second [4,11,12,13,14]. These studies also revealed that 10% of hospitalized RSV-infected newborns may go on to acquire asthma later in life, and that wheezing from lower respiratory tract infections caused by RSV infection could last for a long time [4,11,12,13,14]. However, following a 5-year follow-up period, 50% of individuals with severe Adv-associated pneumonia resulted in the development of PIBO (postinfectious bronchiolitis obliterans) [4,11,12,13,14].

Numerous studies revealed that between 50 and 90% of lower respiratory tract infection cases in children under the age of five were caused by viral pathogens. The majority of viral–bacterial co-infected pneumonia cases affected children younger than 2 years old [2,13,14]. These data were entirely consistent with our study’s findings, which showed that 94 out of 138 cases (68.1%) had bacterial–viral co-infection and 75.4% (104/138) had viral pathogens detected. Children who obtain community-acquired pneumonia frequently have both bacterial and viral co-infection, which exacerbates the illness and raises the death risk. The results of our investigation were fully consistent with the incidence of bacterial and viral co-infection in pneumonia, which can reach up to 68% of hospitalized patients [2,11]. Numerous investigations also revealed that *S. aureus* and *S. pneumoniae* were the most frequently found bacterial pathogens co-infected with viral pathogens [9,12,13]. This finding was consistent with our investigation, as the two bacterial pathogens detected with the highest ratio were *S. pneumoniae* and *staphylococci*.

In the current study, the majority of patients with Adv infection had SpO2 levels below 85% at admission, followed by SpO2 levels between 85% and 90%. Additionally, 71.4% of patients had increased work of breathing with severe chest withdrawal, and 28.6% had chest withdrawal. On the other hand, a significant number of patients in the group infected with RSV had an initial SpO2 level between 85% and 90% (50%), 47.7% had a level between 91% and 94%, and only 2.3% of cases had a level below 85%. When it came to the level of the withdrawal chest, most patients in the Adv-infected group (71.9%) had severe indrawing, but many patients in the RSV group only experienced less severe indrawing chests. Zampoli’s study [15] showed that hypoxemia affected 70.9% of children with pneumonia related to Adv, whereas Li Min Lim’s study [14] showed that respiratory failure affected 67.2% of the children. As a result, when severe Adv-infected pneumonia vs. RSV worsens, it should be recognized and treated right away [3,12,15].

While several of the children in the Adv-infected group of our study received treatment for longer than 30 days, most of the RSV-infected children were hospitalized for less than two weeks. While most patients in the RSV group changed antibiotics just once throughout therapy, the majority of the Adv group required antibiotic changes three times (14.3%). As previously mentioned, the RSV group mostly needed oxygen or cannulas for respiratory assistance, but the Adv-infected group needed NCPAP or other cutting-edge techniques. Children with advanced disease were susceptible to not responding to the first round of antibiotics (cefotaxime or ceftriaxone), which would mean a lengthier course of treatment. In our investigation, every instance with advanced pneumonia had a co-infection with at least one species of bacteria, necessitating the use of antibiotics. In addition, our patients in both groups were readmitted to the hospital; however, the Adv group was the only one to be readmitted three times, with one case progressing to pneumonia after contracting an infection (based on clinical and chest CT scan data, ruling out other variables that could induce interstitial lung damage). A 5-year follow-up study was carried out by Rodriguez [3] on 38 children who were hospitalized in 1998 during an outbreak of Adv pneumonia in Santiago, Chile. According to the study, almost 50% of the cases went on to develop PIBO, and the requirements for oxygen therapy, mechanical ventilation, and ICU (intensive care unit) hospitalization were linked to this complication. While the incidence of pneumonia due to Adv was only 25–12%, serotypes 3, 7, and 14 have the potential to cause fatal necrotizing pneumonia [3,15,16,17,18,19,20,21,22,23]. This could be one of the factors contributing to severe pneumonia linked to Adv in pediatric patients. The small patient population and the fact that this study was carried out in a specialist hospital are its two primary limitations. Additionally, due to the small group sizes of each respiratory virus, we were unable to ascertain the independent effect of each respiratory virus concurrently diagnosed with the illnesses. Lastly, it was not possible to evaluate the effect of co-infection on the long-term effects of RSV and Adv infection.

## 5. Conclusions

Bacterial–viral co-infection pneumonia has a high prevalence in children under 2 years of age, with *S. pneumoniae* infection and RSV being the primary contributors. Severe CAP with wheezing is suggestive of RSV infection, while severe CAP requiring oxygen with a high-grade and prolonged fever, elevated WBC, elevated liver enzymes, and prolonged treatment time suggests Adv in conjunction with drug-resistant bacteria. To aid clinical physicians in stratifying management plans and to help limit the spread of these two viruses, rapid tests and MPL-rPCR to detect RSV and Adv should be indicated in patients with severe CAP requiring oxygen.

## Figures and Tables

**Table 1:** Some clinical characteristics of RSV and Adv among children with pneumonia.

Clinical Features		RSV (*n* = 44)	Adv (*n* = 7)	*p*-Value
Temperature	<38 °C	35 (79.5%)	2 (28.5%)	0.001
	38 °C–38.5 °C	0	1 (14.3%)	
	38.6 °C–39 °C	9 (20.5%)	3 (42.9%)	
	>39 °C	0	1 (14.3%)	
SpO_2_	<85%	1 (2.3%)	1 (14.3%)	0.518
	85%–90%	22 (50%)	3 (42.9%)	
	91%–94%	21 (47.4%)	3 (42.9%)	
Chest indrawing	Indrawing	36 (81.8%)	2 (28.6%)	0.001
	Severe indrawing	8 (18.2)	5 (71.9%)	
Wheezing		34 (77.3%)	5 (71.4%)	0.042
Wheezes—rhonchi		34 (77.3%)	6 (85.7%)	0.008

**Table 2:** The bacterial and viral pathogen detection rate by MPL rPCR.

Bacterial Pathogen	n	%	Viral Pathogen	n	%
*Streptococcus pneumoniae*	69	49.8	Respiratory syncytial virus (RSV)	44	31.9
*MRSA*	18	13.1	Adenovirus (Adv)	7	5.1
*MRSE*	15	10.6	Rhinovirus	12	8.7
*Staphylococcus epidermidis*	6	4.1	Bocavirus	28	20.3
*Hemophilus influenza UT*	12	9	Influenza virus A	1	0.7
*Moraxella catarrhalis*	7	4.9	Parainfluenza virus type 3	21	15.2
*Mycoplasma pneumoniae*	8	5.7	Cytomegalovirus (CMV)	35	25.4
*Chlamydia trachomatis*	18	13.1	Epstein–Barr virus (EBV)	5	3.6
*Burkholderia cepacia*	5	3.3	% is the percentage of pathogen detection in 138 NTA samples collected from patients MRSA: methicillin-resistant *Staphylococcus aureus* MRSE: methicillin-resistant *Staphylococcus epidermidis*		
*Escherichia coli*	26	18.8			
*Klebsiella pneumoniae*	16	11.3			
*Acinetobacter baumannii*	9	6.5			
*Pseudomonas aeruginosa*	9	6.5			

**Table 3:** Laboratory characteristics of severe pneumonia associated with RSV and Adv.

Laboratory Features		RSV (*n* = 44)	Adv (*n* = 7)	*p*-Value
WBC	≤15,000 cell/mm^3^	32 (72.7%)	2 (28.6%)	0.047
	>15,000 cell/mm^3^	12 (27.3%)	5 (71.4%)	
Neutrophil count	<8000 cell/ mm^3^	30 (68.2)	2 (28.6%)	0.064
	≥8000 cell/mm^3^	14 (31.8%)	5 (71.4%)	
CRP	≤35 mg/L	34 (77.3%)	2 (28.6%)	0.012
	>35 mg/L	10 (22.7%)	5 (71.4%)	
Elevated liver enzyme *	Yes	3 (6.8%)	4 (57.1%)	0.00001 **
	No	41(93.2%)	3(42.9%)	
Chest X-ray	Bilateral infiltrates	18 (40.9%)	6 (85.7%)	0.359 **
	One-sided infiltrates	14 (31.8%)	0	
	Consolidation	3 (6.9%)	0	
	Right upper lobe collapse	8 (18.2%)	1 (14.3%)	
	Pneumonia–pleural effusion	1 (2.3%)	0	

Elevated liver enzyme *: ALT > 45U/L and/or AST > 60U/L, **: Fisher.

**Table 4:** Treatment characteristics of severe pneumonia associated with RSV and Adv.

Treatment Characteristic		RSV (*n* = 44)	Adv (*n* = 7)	*p*-Value
Duration *	≤7 days	4 (9.1%)	0	0.168
	8–14 days	26 (59.1%)	1 (14.3%)	
	15–30 days	10 (22.7%)	3 (42.9%)	
	>30 days	4 (9.1%)	3 (42.9%)	
Antibiotic change	1 time	20 (45.5%)	3 (42.9%)	0.18
	2 times	4 (9.1%)	2 (28.6%)	
	≥3 times	2 (4.5%)	1 (14.3%)	
Respiratory support	Oxy/cannula	20 (45.5%)	2(28.6%)	0.619
	HFNC *	1 (2.3%)	0	
	NCPAP	20 (45.5%)	4 (57.1%)	
	ETT ventilation	3 (6.8%)	1 (14.3%)	
Re-hospitalization **	1 time	8 (18.2%)	3 (42.9%)	0.002
	2 times	0	0	
	≥3 times	0	2 (28.6%)	

HFNC: high-flow nasal cannula, *NCPAP*: Nasal Continuous Positive Airway Pressure, ETT ventilation: endotracheal tube ventilation. * Duration is the total number of days that the patient was treated in the hospital. ** Re-hospitalization is hospitalization again because of recurrent pneumonia within 1 month after discharge from the hospital.

## Data Availability

The data that support the findings of this study are available from the corresponding author upon reasonable request.
